# 763 The Cardioprotective Effects of Sildenafil, PJ34, MitoTEMPO, and ZLN005 in the Early After Burn Injury

**DOI:** 10.1093/jbcr/irad045.238

**Published:** 2023-05-15

**Authors:** Ravi Radhakrishnan, Jake wen, Jana DeJesus, Geetha Radhakrishnan

**Affiliations:** University of Texas Medical Branch, Galveston, Texas; University of Texas Medical Branch, Galveston, Texas; University of Texas Medical Branch, Galveston, Texas; University of Texas Medical Branch, Galveston, Texas

## Abstract

**Introduction:**

Cardiac function has been found to decrease within the first 24 hours after burn injury and has been documented to stay depressed for up to 72 hours. Cardioprotective drugs such as sildenafil, PJ34, MitoTEMPO, and ZLN005 have been shown to counteract this dysfunction when administered 1 hour before burn injury. However, there remains limited knowledge on the effects of cardioprotective drugs in the early phase of burn injury.

**Methods:**

Sprague-Dawley rats were divided into 6 groups. In 5 groups, a 60% TBSA scald burn was performed. Cardioprotectors Sildenafil, PJ34, MitoTEMPO, and ZLN005 were administered to 6 rats per group. A control group of 6 unburned rats was maintained in synchrony. Burned and unburned rats were sacrificed after measurement of heart function using echocardiography (ECHO, Vevo2100 echocardiograph). Heart rate (HR), left ventricular ejection fraction (LVEF), fractional shortening (FS), cardiac output (CO), intraventricular septum (IVS), left ventricular (LV) mass, left ventricular wedge pressure (LVWP) and left ventricular internal diameter (LVID) were assessed with the Vevo2100 echocardiograph software.

**Results:**

Compared to sham control, heart rat (HR), diastolic LVID significantly increased at 3 hours post burn. CO, EF, FS, IVS in systole and diastole decreased at 3 hours post burn. LVID after administration of Sildenafil reduced heart rate at 3 hours post burn. ZLN005 restored burn-induced IVS systolic and diastolic decrease at 3 hours post burn. All drugs improved burn-induced EF, FS. Sildenafil and TEMPO recovered CO at 3 hours post burn.

**Conclusions:**

In this study, cardiac function was found to be reduced up to 13 days after initial injury. Sildenafil, ZLN005, and TEMPO were shown to recover cardiac function at 3 hours post-injury, showing improvements in EF and FS. Sildenafil reduced HR and CO while ZLN005 restored burn-induced IVS during systole and diastole. This suggests the cardioprotective utility of these medications if administered early after burn injury.

**Applicability of Research to Practice:**

Will help improve critical care and understanding of cardiac function.

